# Promoting Diversity in the Biomedical Sciences with the Teen Science Ambassador Program

**DOI:** 10.15695/jstem/v7i1.07

**Published:** 2024-04-17

**Authors:** Samantha M. Margherio, Ren Rountree, Jennifer Crooks-Monastra, Bradley F. Brazell, Rodrick Bellamy, Lindsay M. Squeglia

**Affiliations:** 1 Virginia Polytechnic Institute and State University, Blacksburg, VA;; 2 Medical University of South Carolina, Charleston, SC;; 3 University of South Carolina, Columbia, SC

**Keywords:** Diversity, Biomedical Sciences, STEM Education, Community Partnerships, Career Development

## Abstract

Mental health and substance use fields suffer from underrepresentation of racially and ethnically minoritized, first-generation college student, and female members. The homogeny of the current workforce can impede scientific productivity, creativity, and problem-solving in addressing health-related issues. Our team developed the Teen Science Ambassador Program (TSAP) to provide underrepresented minoritized (URM) high school students with science-focused education, research opportunities, and mentoring within their community. The goals of the current study were to describe the logic model and structure of TSAP, provide access to a resource bank to facilitate replication across communities, and present preliminary mixed-methods outcome data to guide development of the program. Qualitative and quantitative results from our first two cohorts (*N* = 18; 89% girls; 72% Black or African American; 22% Hispanic or Latino; 40% of parents did not have a college degree) indicated TSAP contributed to sustained interest, increased confidence, and enhanced sense of belonging in science-related fields, especially those pertaining to mental health and substance use. These findings highlight the program’s promise to facilitate entry and sustainment of URM and female youth within the biomedical sciences. Given the urgent need to promote diversity in the mental health and biomedical workforce, we provide readers with a resource bank to facilitate replication across communities.

## INTRODUCTION

Mental health and substance use are major public health concerns, with evidenced structural and systemic disparities in diagnosis, access to treatment, and outcomes across racial, ethnic, and socioeconomic groups (Burlew et al., 2021; Cook et al., 2017). Yet, the biomedical, clinical, behavioral, and social science fields traditionally tasked with researching and developing treatments to address such concerns suffer from underrepresentation of racially and ethnically minoritized (Hur et al., 2017), first-generation college student (Bettencourt et al., 2020), and female (Carr et al., 2018; White et al., 2021) members. Indeed, despite making up 37% of the U.S. population (U.S. Census Bureau, 2022), racially and ethnically minoritized groups make up just 15% of psychology doctoral recipients, a lower rate than in other biomedical sciences or engineering fields (Hur et al., 2017). Similarly, the percentage of U. S. medical students who are Black or Latino/a is 2–4 times as low as the percentage of U.S. population that are Black or Latino/a, with this underrepresentation being worse in 2019 than in preceding decades (Morris et al., 2021). First-generation students, who are overrepresented within racially and ethnically minoritized groups, are less likely than their peers to pursue degrees in math and science broadly, and more likely to leave college without a degree (Bettencourt et al., 2020). Further, although women may be well-represented at the undergraduate level across science fields (National Center for Science and Engineering Statistics, 2021), they are less likely than men to obtain full professor positions, and they face additional barriers to academic productivity, including within psychology (White et al., 2021). In turn, federal dollars funneled into biomedical research and treatment development are disproportionately distributed to non-Hispanic White principal investigators (Ginther et al., 2011; Hoppe et al., 2019), with such racial/ethnic disparities in funding being especially pronounced among women of color (Ginther et al., 2016).

Collectively, the mental health and substance use workforce severely underrepresents the increasingly diverse patients whom we serve, across both the treatment (Hyde, 2013) and research workforce (Bernard et al., 2013). The cumulative impact of such underrepresentation in our biomedical workforce cannot be overstated as the homogeny of the current workforce impedes scientific productivity, creativity, and problem-solving in addressing mental health and substance use issues (National Institute of Health, 2022). Because these concerns are highly relevant to the context of adolescence (Merikangas et al., 2010), this developmental period may be an ideal time to begin to enhance interest and skills in biomedical sciences via research in these domains.

Strategic community partnerships in science, technology, engineering, and mathematics (STEM) education represent a key strategy to promote inclusion of underrepresented, minoritized (URM), and female individuals within the biomedical, clinical, behavioral, and social sciences (Margot and Kettler, 2019; National Science and Technology Council, 2021). Such partnerships can overcome challenges faced by under-resourced school districts (Margot and Keller, 2019) to provide hands-on STEM learning experiences at a time when high school students are exploring and solidifying career options (Porfeli and Lee, 2012; Sahin et al., 2017). Indeed, youth report hands-on learning experiences as important to their sustainment of STEM interest (VanMeter-Adams et al., 2014), and such early STEM exposure and socialization can facilitate later degree persistence among URM students (Bettencourt et al., 2020). Additionally, community partnerships among school districts and biomedical research institutes offer opportunities for students to gain access to knowledgeable professionals and near-peer mentors. For URM and female youth, such mentorship leads to increased confidence and professional skills (Tenenbaum et al., 2014), sustained intent and support to pursue STEM degrees (Fuchs et al., 2016; Schultz et al., 2011), and role models for desired career pathways (Tenenbaum et al., 2017).

Our team developed the Teen Science Ambassador Program (TSAP) via strategic community partnerships to provide URM and female high school students (i.e., ambassadors) with education, hands-on research experience, and mentoring within mental health and substance use fields. The overarching aim of TSAP is to promote diversity within the biomedical, clinical, behavioral, and social sciences workforce by preparing underrepresented high school students with research knowledge and professional development opportunities to facilitate future careers in mental health and substance use fields. The goals of the current study were to describe the logic model and structure of TSAP, provide access to a detailed program overview and a resource bank to facilitate replication across communities, present mixed-methods pilot data from the initial two cohorts to demonstrate TSAP’s preliminary outcomes, and discuss lessons learned to inform future iterations of TSAP.

## METHODS

### Logic Model.

Table 1 outlines the logic model underlying TSAP, spanning from the community resources and students that comprise the program to the anticipated outcomes for URM ambassadors. Table 2 displays the three-phase structure of the TSAP designed to meet the overarching aims of the program. We encourage interested readers to access our TSAP resource bank (https://sites.google.com/view/tsap-resources/home) for additional details regarding program coordination and content, including specific details to allow for program replication. To shift the onus of science-based education from often under-resourced schools to the broader community, the program is coordinated through a research collaborative housed in an academic medical center and funded via grants from the National Institute of General Medical Sciences. The research collaborative partners with an Advisory Committee and community partners to enhance program feasibility and acceptability. The program aims to build high school students’ communication skills, research literacy, and knowledge about opportunities for URM in mental health and substance use fields by providing mentored engagement in research and career experiences. Such proximal outcomes are hypothesized to translate to increased confidence, self-efficacy, maintained interest in scientific fields, and pursuit and persistence of degrees and careers related to biomedical sciences, especially those pertaining mental health and substance use.

### Program Coordination.

TSAP is organized and facilitated by a faculty program director and a full-time bachelor’s-level program coordinator employed within an academic medical center in the southeastern United States, specifically within a research collaborative focused on adolescent mental health and substance use. The director oversees the program and secures federal funding to support the program. The coordinator is responsible for facilitating community partnerships, recruitment and selection of ambassadors and mentors, developing the program structure, scheduling, and coordinating ambassadors’ transportation and onboarding materials. Administrative support is available from the academic medical center (e.g., information technology, human resources, library services) and supported by indirect funds from grants. Additionally, we partner with a consulting evaluation team to facilitate program evaluation, including coordination and analysis of quantitative and qualitative feedback.

### Community Partnerships and Advisory Committee.

Community partnerships in TSAP are facilitated in part via a Community Advisory Committee, consisting of high school teachers, principals, and members of parent advisory groups. Members of the Committee are nominated and elected by program or board members. Priorities of the Advisory Committee include (1) advising the research team on program activities, curriculum development, and selection of students; (2) assisting in any grievances related to the TSAP (none to date); (3) providing advice on fiscal issues; (4) identifying funds and resources to broaden program impact; and (5) assessing overall program progress.

TSAP curriculum is designed and reviewed by local high school teachers, including a STEM teacher serving as our Curriculum Advisor, to ensure alignment with educational standards and priorities. Other faculty and staff (e.g., school counselors) at community high schools also play a role by connecting TSAP ambassadors with career opportunities such as job shadowing and college preparation, and coordinating program logistics (e.g., transportation).

### Ambassadors.

A total of 18 ambassadors have participated in the TSAP (six in the first cohort; 12 in the second cohort). Ambassadors were in grades 10 or 11 representing 10 high schools, with 39% of ambassadors coming from Title 1 (designated low-income) schools. Ambassador demographic information is presented in Table 3.

#### Recruitment and Selection.

Recruitment of URM and female youth is conducted via community outreach (e.g., representation at area STEM festivals), information sessions hosted at area schools, advertisement and guest lectures with school STEM clubs, and attendance at school events. TSAP recruits individuals from National Institute of Health-defined underrepresented groups, defined as racial and ethnic groups underrepresented in biomedical research, individuals with disabilities, individuals from disadvantaged backgrounds, and women (National Institute of Health, 2022; see https://diversity.nih.gov/about-us/population-underrepresented). We encourage individuals from underrepresented groups to apply if they meet the following criteria: (1) maintain any interest or curiosity about STEM; (2) have limited access to other STEM opportunities; and (3) are interested in the program. Note that evidence of academic success (e.g., GPA) is not considered in the application, and we encourage applications from individuals without prior involvement in STEM opportunities. Individuals complete an application related to their leadership and job experience and future goals. Guardians must provide consent for the individual to engage in the program.

Applicants then enter a two-step interview process. Each candidate is asked the same performance-based questions to assess their interest in biomedical sciences, potential for leadership, and enthusiasm for the program. Prior to the interview, the program coordinator shares online resources with students to allow them to prepare for the interview, engages applicants in a mock interview, and provides feedback on their performance. Applicants then meet with the program director who asks the same questions and completes a quantitative rating scale based on the applicant’s performance. The program director meets with senior mentors to select applicants for the program based on the interview rating scale and qualitative comments, with consideration for applicants’ potential when provided with opportunities possibly inaccessible elsewhere. Regardless of whether applicants are invited to participate in the program, all are provided with materials related to local STEM training and programming to continue to foster interest. Thus, a unique aspect of the TSAP recruitment structure is that all applicants, regardless of selection, receive interview experience and feedback, and receive information for additional STEM opportunities.

#### Senior and Near-Peer Mentors.

Rather than relying on the prevailing dyadic mentoring model, TSAP leverages a mentoring triad in which each ambassador is paired with one near-peer mentor and one senior mentor. The mentoring triad has demonstrated benefits for all members of the group, including normalizing struggles and bolstering scientific identities among ambassadors who have access to peers from similar backgrounds (see Markle et al., 2022) and boosting the scientific identity, self-efficacy, and sense of belonging among near-peer mentors (Trujillo et al., 2015). Senior mentors are predoctoral students, postdoctoral fellows, and faculty within a research collaborative focused on adolescent mental health and substance use. All senior mentors are involved in research and have experience with research mentorship of high school students. Senior mentors meet for a two-hour mentor training to review expectations for the program. They also complete the Mentoring Competency Assessment (MCA; Fleming et al., 2013) prior to and after the program as a self-assessment of their mentor competencies.

Near-peer mentors are selected from former ambassadors who completed the first phase of the program (see below). Because all near-peers are former ambassadors, they are individuals from groups that are underrepresented in the biomedical and clinical sciences. All near-peer mentors undergo a mentorship training course (see Mentoring Training below).

### Program Outline.

#### Phase One: Didactics.

First, ambassadors engage in weekly didactic lessons that introduce clinical research, emphasize diversity within clinical research, and review skills necessary for a career in the biomedical science field (see Table 2). Curriculum for the program was informed by the Common Core and Next Generation Science Standards. The curriculum was developed iteratively through collaboration of the program faculty and staff, consultation with high school teachers and our Curriculum Advisor, the Community Advisory Group, and pilot testing.

Ambassadors engage in a triadic mentoring program throughout this 16-session phase with a senior mentor and a near-peer mentor. Whereas senior mentors are often involved in a teaching and guiding role, near-peer mentors serve as both teachers and participants through Phase One. For example, near-peer mentors engage in all of the didactic lessons, and they are invited to speak during certain lessons, including sharing their experiences thus far with navigating college applications and financial aid and sharing their own research projects from Phase One. A key focus is on building ambassadors’ confidence and self-identity within STEM, and therefore all mentors are encouraged to adopt a motivational, supportive mentoring style that also addresses personal growth and barriers to confidence.

Senior mentors, with assistance from near-peer mentors, guide ambassadors through an inquiry-based research project, pursuing a scientific research question related to substance use and/or mental health. Mentors help ambassadors identify a research question by asking, “If you could ask any question about mental health and substance use, what would it be?” and then further molding the question to something addressable with a literature review. Ambassadors learn to conduct literature reviews, weigh evidence, form conclusions based on their research, summarize their research in a poster, and disseminate their findings to professionals in the field during a mini-symposium at the conclusion of Phase One.

Hands-on research experience for youth from URM backgrounds is believed to be associated with pursuit of scientific careers (Linn et al., 2015), yet these youth likely also require additional structural and logistical support for navigating their pursuit of scientific careers (Rocha et al., 2022; Tilghman et al., 2021). Therefore, didactic lessons also focus on college readiness, applications, and financial aid, as well as broadly exposing youth to scientific career options via tours of the academic medical center and labs and guest speakers. Mentors also provide guidance and support for professional development, such as discussing college options, reviewing application materials, engaging in mock interviews, modeling effective networking, and connecting ambassadors with opportunities that meet their interest. A syllabus, included detailed weekly lessons, is provided in the in our TSAP resource bank.

#### Phase Two: Hands-on Research Internships.

Phase Two is focused on continued networking and exposure to other biomedical science-related opportunities as ambassadors engage in a research internship tailored to their interests. Ambassadors are invited to serve as paid interns for 100 hours on NIH-funded research projects to facilitate work-based learning strategies. Ambassadors prepare application materials, apply, and interview for internship positions to further build career-building skills and a sense of self-efficacy for job attainment. Interns receive a competitive hourly wage and can receive support for travel to and from the office, and schedules are flexible to accommodate school, family, and other extra-curricular activities of the interns.

#### Phase Three: Near-Peer Mentoring.

For Phase Three, graduates of Phase One are invited to serve as near-peer mentors for upcoming TSAP ambassadors to increase their self-identification as scientists and foster professional skills. Near-peer mentors utilize the trainings and clinical research knowledge from Phase One as they mentor incoming ambassadors and actively participate in didactics as a junior-level scientist. Near-peer mentors are supervised by senior mentors who provide guidance and training in mentorship skills, including communication, establishing professional relationships, and cultural humility in mentorship.

### Mentoring Training.

During a six-hour hybrid (self-paced online and face-to-face) training, the near-peer mentors receive training developed utilizing the Ready to Go: Mentor Training Toolkit (Bottomley and Frendo, 2012). This toolkit was designed to give mentors the skills they need to build successful relationships with mentees. Additionally, near-peer mentors are provided with the Peer Mentoring Handbook created for mentors ages 12–22 (The Mentoring Partnership of Southwestern Pennsylvania, 2014) which gives mentors an overview of what to expect when serving as a peer mentor to a younger student. The Handbook also includes recommended practices for establishing and maintaining a quality, high impact peer mentoring relationship. Near-peer mentors engage in self-assessments of their own mentoring competencies at the outset and conclusion of Phase One using the MCA (Fleming et al., 2013). The purpose of the MCA is to highlight near-peer mentors’ areas for growth and stimulate conversations between near-peer and senior mentors to shape training needs.

### Ongoing Support.

All ambassadors have access to our alumni electronic mailing list, which includes career opportunities for URM and women in science and highlights alumni of the program who have gone on to earn higher degrees and positions in biomedical sciences. Ambassadors are invited to join biannual ambassador alumni group virtual meetings, which include networking events with faculty, alumni, and staff. As students progress through their education and career, they can be enrolled in other mentoring programs focused on college and post-baccalaureate students.

#### Program Changes.

Programmatic changes from Cohort One (spring 2022) to Two (fall 2022) included: (1) changes to the research component, (2) addition of near-peer mentors, and (3) transition to in-person sessions. Cohort One gave a formal talk to disseminate research findings at the conclusion of Phase One, whereas Cohort Two engaged in a conference-style poster session. Cohort Two also experienced the addition of near-peer mentors. While this program was originally created to be in-person, Cohort One completed Phase One entirely remotely due to COVID-19. The 16-session curriculum for Cohort Two was delivered via 10 weekly in-person sessions and additional online videos and assignments to facilitate exposure to the biomedical science environment and comfort in mentoring relationships.

### Addressing Barriers.

TSAP was iteratively developed to address logistical and structural barriers to STEM education commonly faced by URM youth. All in-person programming is hosted after-school and the program coordinator works with families and schools to coordinate free transportation for ambassadors to and from meetings. Meals are provided during these meetings. Didactic materials are offered online when possible, and the program coordinator works with schools and families to ensure online materials are accessible for teens. Additionally, ambassadors earn a competitive wage for participation in all phases of the program. Notably, provision of competitive wages during training has been identified as a key element of successful research training programs for individuals from URM backgrounds (Tilghman et al., 2021). These programmatic costs are possible via grant funding and in-kind donations.

### Program Evaluation.

Both qualitative and quantitative evaluations were used to collect data from TSAP stakeholders (ambassadors, near-peers, senior mentors, and project leadership team). Focus group protocols were designed, and survey instruments were selected to assess the degree to which the proximal goals of TSAP were achieved: sustaining and expanding interest in biomedical science, enhancing sense of belonging and identification with biomedical sciences among ambassadors; enhancing ambassadors’ self-efficacy for learning and doing science; and engaging stakeholders in an overall positive program experience.

#### Focus Groups.

An external evaluation team conducted focus groups with ambassadors, near-peer mentors, senior mentors, and the project leadership team following the completion of Phase One of each cohort. The purpose of the focus groups was to learn about participants’ experiences in and perceptions of TSAP. Specifically, focus groups provided data on ambassador’s confidence, self-efficacy, STEM identity, STEM interest, career plans, and perceived changes in skills or knowledge. Fifteen of 18 ambassadors, all five near-peer mentors, and 10 of 14 senior mentors participated in the focus groups. Qualitative data collected from the focus groups was transcribed and analyzed through memos (Saldaña, 2016) for emerging themes and used as formative and summative feedback.

#### Teen Ambassadors Outcomes Survey.

The Teen Ambassadors Outcomes Survey was developed by the project team using a combination of scales from validated instruments to assess ambassadors’ sense of belonging, identity, interest, and self-efficacy in biomedical sciences. The survey included the STEM – Belonging, Growth Mindset, and Identity scale (Kricorian et al., 2020), a 7-item Likert-scale measure assessing the degree to which one agrees that they belong in the STEM field. Additionally, we included three items that assess ambassadors’ beliefs that STEM careers are accessible for them (Kricorian et al., 2020). The Self-Efficacy for Learning and Doing Science Scale (Phillips et al., 2014) is an 8-item Likert-scale measure that assesses the degree to which one has confidence in their ability to participate in science. The STEM Career Interest Survey (STEM-CIS; Kier et al., 2013) is an 11-item Likert-scale measure of teens’ interest in STEM classes and careers.

The Outcomes Survey was administered to ambassadors and near-peer mentors prior to and after participation in Phase One via REDCap (Harris et al., 2009). Responses are available for all but one ambassador who did not complete the survey after several contact attempts. Given these small sample sizes, quantitative data were evaluated with descriptive statistics and pre/post Hedge’s *g* corrected for small sample size (Hedges and Olkin, 1985).

## RESULTS

### Program Engagement.

Among the 18 ambassadors, 15 engaged in 100% of Phase One sessions, and the remaining three engaged in 80% of sessions. All six ambassadors from Cohort One continued in Phase Two and completed 100-hour internships, and nine (75%) of Cohort Two ambassadors have initiated internships. Five of the six Cohort One ambassadors became near-peer mentors for Cohort Two. These five attended an average of 82% of Phase One sessions as near-peer mentors. As of this writing, eight of the 12 Cohort Two ambassadors became near-peer mentors for the subsequent cohort (ongoing).

### Interest in Biomedical Sciences.

Qualitative and quantitative feedback indicated most ambassadors maintained or increased their interest in biomedical sciences (see Tables 4 and 5). Qualitative data indicated that, although many ambassadors entered the program with a high degree of interest in careers pertaining to science (*M* = 4.06 on a 5-point scale; *SD* = 1.03), the program helped solidify their career choice (e.g., “[STEM] is actually like what I want to do as a career now”; “it allowed me to gain insight and secure whether I want to do this or not”), broaden their knowledge of options in STEM (e.g., “Before the TSAP I only really thought about nursing as a career for myself, but now I’m looking more to psychology and the careers that can come from that major…”), or enhance their passion for these fields (e.g., “… it fueled my curiosity and the entire project…it just fueled my interest(s)”). Ambassadors specifically noted the utility of learning about the brain, being able to be on an academic medical center campus, MRI tours, and guest lecturers from diverse fields as helpful for fueling their interest in biomedical science career fields. Most ambassadors (71%) reported sustained interest in science careers following Phase One, with a smaller portion (24%) reporting increased interest in scientific careers.

### Sense of Belonging and Identity with Biomedical Sciences.

Focus group feedback across ambassadors, near-peer mentors, and senior mentors indicated that mentoring was a powerful component of TSAP that contributed to growth in ambassadors’ sense of belonging and identity with biomedical sciences (Table 5). Comments reflected the benefit of the mentoring triads and therefore enhanced exposure to a mentors from diverse backgrounds in building sense of belonging among ambassadors (e.g., “I feel growing up there weren’t as many like figures we could relate to … now you’re able to talk to someone who is able to understand your struggles and [see] how they’ve been able to deal with it and how they are able to… manage their time and like be successful in their fields despite you know, their backgrounds”).

Per outcome surveys, ambassadors left Phase One with an improved sense that they had a role model within biomedical sciences (*g* = .32; see Table 4). They also generally reported an increased sense of belonging in science (*g* = .27), with most ambassadors (71%) reporting improved scores on the STEM belonging measure. Cohort Two (*g* = .33) reported slightly greater improvements in their sense of belonging in science than those in Cohort One (*g* = .16). Despite the increased sense of belonging, Cohort One reported an increased sense of barriers to accessing STEM education and careers following Phase One (*g* = .32), whereas Cohort Two had negligible effects on their sense of barriers (*g* = −.08). Less than half of ambassadors reported the same (6%) or fewer barriers (41%) to STEM fields following Phase One.

Notably, preliminary evidence indicated that our program’s unique emphasis on mentoring triads and use of near-peer mentors further solidified the sense of belonging in STEM for ambassadors who continued as near-peer mentors for our second cohort. Near-peer mentors rated the degree to which they identify themselves with a STEM professional on a seven-point scale (STEM-PIO-1; McDonald et al., 2019). Most near-peer mentors rated themselves between a five and six, although one near-peer with interests outside of STEM rated themselves at a three. The average was 5.2, which was higher than the 3.9 average they reported at the end of their semester as ambassadors. This provides additional evidence to suggest that continued involvement in the TSAP through internship experiences and serving as a near-peer mentor may further enhance STEM identity.

### Self-Confidence and Self-Efficacy for Science.

Ambassadors grew in confidence, particularly related to speaking about STEM content or using STEM terminology. One senior mentor noted that ambassadors “became more confident in talking about things related to science specifically.” The ambassador’s fluent use of a medical term provided evidence for the mentor that “we are solidifying different terms that are used in science” and that the mentee had grown in understanding of the terms and how they can be applied. These benefits appeared to continue for those ambassadors who continued as near-peer mentors, with a senior mentor noting, “She also was always confident, but I remember when she first met the mentees. She went up to them and was like, this program was so great, and I learned all these things and [was] just talking like she was one of the mentors! Just super confident and like she knew everything about the program. It was just awesome to see her growth because I could not have seen her doing that last year.”

A noteworthy example of TSAP’s impact is from an ambassador whose high school science teacher attended the research presentations and spoke to the senior mentor. According to the mentor, the high school teacher witnessed growth in the ambassador’s “confidence, especially in science, and that has actually come along with improved grades in her chemistry course as well.”

On the outcome survey, ambassadors reported an increased sense of self-efficacy in science (*g* = .17), with about half of ambassadors (53%) reporting improved scores on the science self-efficacy measure. There appeared to be a trend such that Cohort One (*g* = .23) reported greater improvements in their sense of self-efficacy in science than Cohort Two (*g* = .08). Despite these small gains in self-efficacy, Cohort Two reported slightly decreased comfort in talking to others in science careers (*g* = −.20), whereas Cohort One reported moderately increased comfort in talking to others in science careers (*g* = .50). Still, across both cohorts, most youth remained stable (59%) or increased (24%) in their comfort talking to others in science careers.

### Overall Program Experience.

Findings synthesized across focus groups suggest that (1) TSAP successfully engaged students in research experiences; (2) the program was enhanced by the addition of near-peer mentors in Cohort Two; (3) TSAP provided high-quality, relational mentoring that supported students as developing researchers; and (4) ambassadors expressed interest in STEM and grew in confidence because of program participation. Table 5 includes quote excerpts reflecting these themes. Additionally, most (72%) ambassadors shared that they did not have a mentor before starting our program. After completing Phase One of the program, 100% shared they have a mentor. Further, 100% of ambassadors said they would recommend TSAP to their friends.

### Areas for Continued Program Improvement.

Focus group findings suggest three recommendations for ways in which the TSAP program can continue to develop and improve: (1) structuring the poster presentations; (2) clarifying the near-peer role; and (3) suggestions for broader exposure to the biomedical sciences. Poster presentations may be structured through setting expectations, scaffolding public speaking, and including a brief presentation prior to the poster session. Near-peer mentors shared experiencing some times they felt unnecessary or unsure what to do. Senior mentors also noted a challenge with balancing time and attention between the peer mentor and ambassadors. Both near-peer and senior mentors reported the training and mid-program check-in meetings were helpful and recommended continuing these meetings. All stakeholders suggested increasing the number of panels and inviting guest speakers from diverse areas of biomedical sciences outside the expertise of the senior mentors.

## DISCUSSION

Enhancing the inclusion of URM and female individuals in biomedical fields requires multiple interwoven strategies to address the systemic and structural barriers faced by such individuals along the pathway to a biomedical career (National Science and Technology Council, 2021). The TSAP is just one such strategy that leverages community partnerships to provide access to meaningful hands-on learning experiences and mentoring opportunities for URM and female high school students interested in biomedical fields related to mental health and substance use. In this paper, we provided detailed information about the TSAP infrastructure, processes, and outcomes in hopes of inspiring replication across communities. Our early findings from focus groups and outcome surveys demonstrate proof of concept that the TSAP contributed to development of mentoring relationships, maintained interest, increased sense of belonging, and improved self-efficacy and self-confidence in biomedical sciences among URM adolescents. These domains are hypothesized as key ingredients in consistent pursuit of biomedical science careers (Dorsen et al., 2006). Efforts are underway to continue following these youth to identify the degree to which proximal outcomes translate to educational and occupational attainment in the biomedical sciences.

Although quantitative and qualitative findings related to the ambassadors’ interest, sense of belonging, and self-efficacy are promising, the slight increases in their sense of STEM accessibility barriers are noteworthy. Indeed, 53% of ambassadors reported a greater sense of barriers to STEM education following Phase One, with these adverse effects being more prominent in our inaugural, virtual cohort than our second cohort which was delivered predominantly in-person. The three items on this scale inquire about adolescents’ perceptions of the expense, difficulty, and duration of schooling for STEM careers. Of note, this scale was the only one in the outcomes survey to include entirely reverse-scored items, and thus such adverse findings may simply reflect response biases. Alternatively, it may be that TSAP increased awareness of the cost and endurance often required for biomedical science careers without providing adequate support and resources to overcome these barriers. One strategy to address this challenge came from our focus groups in which ambassadors requested additional exposure to alternative biomedical career pathways rather than exclusive exposure to doctoral-level senior mentors in mental health and substance use fields. Additional next steps to reduce adolescents’ barriers to STEM education include further diversifying the speakers and mentors within the program in terms of cultural diversity and career fields as has been done in other programs (Rocha et al., 2022), engagement with caregivers in college financial planning sessions (Perna, 2002), and provision of ongoing college preparatory resources through graduation (Morgan et al., 2015; Rocha et al., 2022).

### Lessons Learned.

Feedback from focus groups identified concrete suggestions for improving the program’s next iteration, which is currently underway. The removal of a formal research presentation and transition to a conference-style poster session for Cohort Two’s graduation ceremony may have inhibited their sense of growth in comfort talking about science. For our third cohort, we therefore focused an in-person didactic on modeling and practicing research presentations, and we included both a formal research presentation and a conversational poster session during the graduation ceremony. Feedback also led to the addition of didactic sessions of panels of speakers from diverse backgrounds, both in terms of sociodemographic and career backgrounds, to broaden exposure to alternative biomedical science careers. To further clarify the role of near-peer mentors, we developed a weekly checklist for near-peer mentors that included a goal for the week (e.g., “Near peer mentors should help their ambassador find at least two citations that are relevant to their research poster,” “Near peer mentors should share at least two resources with their ambassadors about preparing for college”). We will continue to monitor mixed-method outcome data to identify the impacts of such changes and identify areas for continued improvement. Given findings related to increased perception of barriers, another next step for future cohorts includes increasing guardian/family involvement to develop college financial action plans with family input, such as the addition of family panels.

Additionally, our team has learned that one full-time program coordinator position is insufficient in facilitating our expanding program across all three phases and follow-up, and funding is needed to support more staff time to support the program. In our team, we were able to utilize funds from our internal budget to support a part-time staff member whose duties involved co-coordination of the TSAP, development of new community partners, and strategic efforts with current partners to further prepare ambassadors for college within their academic setting.

### Next Steps.

These findings represent preliminary proof of concept for the TSAP in producing proximal improvements in high school students’ interest, sense of belonging, and self-efficacy for biomedical sciences. If such proximal outcomes are found to translate to pursuit and persistence in mental health and substance use fields, program scalability will be an important issue. Of note, although our program focuses on mental health and substance use fields, we imagine program structure and content could be translated to match the expertise of other biomedical science teams. To bring the much-needed change in diversifying the biomedical fields, much larger numbers of program participants than would be possible through our institution alone would be needed. The TSAP is a time- and resource-intensive program currently funded via multiple cycles of grant support. The indirect and direct costs of the program would require commitment of entities such as large businesses and/or government agencies to promote sustainment and scalability.

Given the resources needed to produce this program, a potential next step could include paring down program components to enhance scalability. Indeed, many aspects of the TSAP that we believe to be unique, such as the inclusion of near-peer mentors and the addition of a paid internship, are extremely time- and resource-intensive. Yet, our current evidence and prior research (see Markle et al., 2022; Tilghman et al., 2021) suggest that these aspects may add to the benefits of the program, although research is needed that helps identify which elements are key to programmatic success. We, and others (e.g., Tilghman et al., 2021), argue that the immense need to diversify the biomedical science fields require equally immense resources, including expanded grant funding to support such intensive programming and cultural shifts to value diversity in the sciences as equal to other scientific advances. In the interim of such financial and cultural shifts, it may be necessary to reserve such intensive program components for scientific fields with particularly poor representation, including the mental health and substance use fields where such underrepresentation can have immediate impacts on the patients whom we serve (Bernard et al., 2023; Hur et al., 2017).

### Limitations.

Findings from TSAP represent short-term outcomes from a small sample of ambassadors, and therefore these preliminary results may not be generalizable. These inaugural cohorts were conducted in the context of the COVID-19 pandemic, which required flexibility in terms of delivery format as well as barriers to building community partnerships for internships. Additionally, our program is for students already interested in biomedical sciences and we do not have a comparison group. It is therefore unclear how our findings speak to the added benefit of TSAP compared to a control condition or whether TSAP could be used to promote interest in biomedical fields among URM and female youth with limited interest at the outset. Finally, we do not have qualitative data to further investigate and explain why youth reported increased STEM barriers following TSAP involvement, but will collect this data in subsequent cohorts.

### Conclusion.

Findings from the initial two cohorts offers proof of concept that the TSAP is one method to leverage community partnerships for the promotion of biomedical science interest, sense of belonging, confidence, and self-efficacy among URM and female high school students. This program is therefore a promising strategy for addressing the urgent need to promote diversity in fields tasked with addressing mental health and substance use needs in the population. We hope to inspire other academic medical centers and research teams to utilize our resource bank (https://sites.google.com/view/tsap-resources/home) and program outline to develop their own programs to address this urgent need.

## Figures and Tables

**Table 1.** TSAP Logic Model.

Goal: The overarching goal of TSAP is to promote diversity within the biomedical sciences workforce by preparing underrepresented high school students with scientific knowledge and professional development opportunities to facilitate and encourage continued future careers in substance use research.			
Inputs and Resources	Summary of Program Activities	Anticipated Proximal Outcomes	Anticipated Distal Outcomes
Funding Science Education Partnership Award from the National Institute of General Medical Sciences	Academic Medical Center Faculty & Staff Secure program funding Establish Advisory Committee Advertise program Recruit and select 12 teen ambassadors from diverse backgrounds each year Develop and revise curriculum Establish internship opportunities Train senior and near-peer mentors Facilitate mentored relationships	Ambassadors will experience: sustained and expanded interest in biomedical sciences increased sense of belonging in and identification with STEM increased self-efficacy for engaging with scientific content increased self-efficacy for developing and researching ideas enhanced knowledge of diversity in STEM enhanced knowledge of research fields increased awareness of financial supports for pursuit of biomedical degrees improved communication skills increased research literacy	Ambassadors will: earn degrees and/or professional certificates in biomedical science areas. have increased employability in biomedical careers, particularly in substance use and mental health fields. become biomedical professionals, leading to an increase in diversity of scientists and STEM mentors. contribute to increasing public health literacy, specifically in the substance use and mental health fields. mentor other students in the biomedical sciences, with a focus on increasing diversity in the field.
Academic Medical Center Faculty & Staff Faculty Director Program Coordinator Senior Mentors Office of Diversity and Inclusion Administrative support			
	Community Partners Develop, review, and revise curriculum Encourage teens from diverse backgrounds to apply Engage in program curriculum as guest speakers, audience		
Consulting Evaluation Team			
Community partnerships County School District Administrators Teachers Counselors STEM Education Centers Teacher partners for curriculum alignment and review Community Advisory Committee High School Teachers Principals Parents	Ambassadors Apply and interview for TSAP Engage in 16 sessions and coursework in topics including clinical research, scientific communication, diversity, cultural awareness, and financial literacy (Phase 1) Engage in mentored research project (Phase 1) Present research to an audience (Phase 1) Develop academic CV, apply and interview for competitive biomedical science internship (Phase 2) Engage in 100 hr paid internship (Phase 2) Train to become near-peer mentors (Phase 3) Join alumni group meetings and mailing list		
Teen Science Ambassadors			
Near-Peer Mentors			

**Table 2.** TSAP Phase Structure.

Phase One: Didactics and Research Project	Phase Two: Internship	Phase Three: Serve as Near-Peer Mentors	Ongoing Support
Didactics 16 didactic lessons, delivered over 10 weeks	Develop CV Engage in mock interviews Interview for STEM internships Engage in 100-hr paid internship in biomedical sciences	Engage in 6-hr mentor training in: Building mentoring relationships Setting boundaries Communication Youth development and cultural humility Engage in self-assessments of one’s own mentoring competency Serve as a near-peer mentor for a new ambassador during Phase One, while being supervised and supported by a senior mentor	Receive mailing list for career opportunities in biomedical sciences Join biannual alumni virtual meetings Enroll in other mentoring programs for college and post-baccalaureate students
*Example topics:* Campus scavenger hunt MRI tour and neuroscience models Intro to clinical research Adolescent substance use Ethics in clinical research Research literature reviews Celebrating diversity in research Disseminating research Interviewing in STEM fields College readiness in STEM College financial aid			
	*Example internships:* Observing clinical research visits Assisting with recruitment for clinical research studies Creating/updating lab websites Presenting at local high schools and community groups Presenting at weekly lab meetings Disseminating research findings via social media		
			*Examples of opportunities alumni have accessed:* Three alumni presented at a regional conference celebrating female mentors Two alumni enrolled in additional mentoring programs within the academic medical center
Mentored Research Project Develop a research question Identify credible resources Create a poster to disseminate findings Present posters at an internal symposium			
*Example research questions:* Is alcohol use disorder affected by genetics? Are teen substance use and mental health related? How is the adolescent brain affected by substance use? Is teen substance use related to social media?			

**Table 3.** TSAP Phase One: Demographics and Quantitative Outcomes by Cohort and Overall.

Demographics	Cohort 1 (n = 6) N (%)	Cohort 2 (n = 12) N (%)	Total (N = 18) N (%)
*Gender*			
Boys	1 (17%)	1 (8%)	2 (11%)
Girls	5 (83%)	11 (92%)	16 (89%)
*Race*			
Black/African American	4 (67%)	9 (75%)	13 (72%)
White/Caucasian	2 (33%)	2 (17%)	4 (22%)
Not listed	0 (0%)	1 (8%)	1 (4%)
*Ethnicity*			
Hispanic/Latino/Spanish	3 (50%)	1 (8%)	4 (22%)
*Parent education*			
High school or less	3 (50%)	4 (33%)	7 (39%)
Some college	0 (0%)	2 (17%)	2 (11%)
Bachelor’s degree	1 (17%)	4 (33%)	5 (28%)
Graduate degree	2 (33%)	1 (8%)	3 (17%)
Unknown	0 (0%)	1 (8%)	1 (6%)

**Table 4.** TSAP Quantitative Outcomes Overall and by Cohort.

Outcomes	Cohort One (*n* = 6)			Cohort 2 (*n* = 11)			Total (*N* = 17)			Overall Direction of Change		
	Pre *M(SD)*	Post *M(SD*)	*g*	Pre *M(SD)*	Post *M(SD)*	*g*	Pre *M(SD)*	Post *M(SD)*	*g*	Increased (%)	Stable (%)	Decreased (%)
Interest in Science Careers (1–5)	4.17 (1.17)	4.50 (.84)	.21	4.00 (1.00)	4.09 (1.04)	.07	4.06 (1.03)	4.24 (.97)	.16	24%	71%	6%
Have a Role Model in Science (1–5)	3.33 (1.63)	4.00 (1.55)	.27	3.09 (1.14)	3.45 (1.21)	.26	3.18 (1.29)	3.65 (1.32)	.32	41%	41%	18%
Comfort Talking to Others in Science (1–5)	4.17 (1.17)	4.83 (.41)	.50	4.27 (.65)	4.09 (.83)	−.20	4.24 (.83)	4.35 (.79)	.13	24%	59%	18%
Sense of Belonging in Science (1–6)	4.33 (1.17)	4.62 (1.13)	.16	4.47 (.44)	4.68 (.59)	.33	4.42 (.75)	4.66 (.79)	.27	71%	12%	18%
STEM Accessibility Barriers (1–6)*	3.28 (1.39)	4.00 (1.56)	.32	3.06 (.98)	2.97 (.82)	−.08	3.14 (1.10)	3.33 (1.20)	.15	53%	6%	41%
Science Self-Efficacy (1–5)	3.94 (.76)	4.17 (.49)	.23	3.66 (.45)	3.71 (.41)	.08	3.76 (69)	3.87 (.48)	.17	53%	0%	47%

Note:

* higher scores on the STEM Accessibility Barriers scale indicated more perceived barriers to STEM education and careers. For all other scales, higher scores reflect improved beliefs and attitudes toward science. Values in parentheses represents range of scores. Hedge’s g reflects pre-post effect sizes, corrected for small sample size.

**Table 5.** TSAP Qualitative Feedback.

**Overall Program Experience**	*“I really love my mentor. She gave me a personal level of a bond because she explained her whole pathway, and she got to know us not only as a mentor but as a person as well. So, it was like she really knew who we were and what our goals in the future were, and she helped us achieve what we wanted to achieve.” (A)*
	*“She’s [mentor] always provided like the best research links …always given like the best feedback, so when I’m like, oh I’m not sure of this, she would not only give an answer, but she would go in-depth and explain it.” (A)*
	*It’s just a good program. You don’t only gain knowledge in like the STEM field, you learn knowledge like how to finance money, how to get up and do presentations, how to do a speech, and you just have a really good support network, and you know people that will be able to help you.”(A)* *“…the experience of coming onto campus, walking into the office, getting a badge, I love my badge …I got a little more social since working here, especially since presenting my project at graduation. I am an extremely shy person; I hate public speaking. I think I have gotten better at it. I have gotten better at networking …I am seeing myself grow a lot during my internship.” (NPM)*
**Interest in Biomedical Science**	*“Before the TSAP I only really thought about nursing as a career for myself, but now I’m looking more to psychology and the careers that can come from that major. And so, it’s just broadened my perspective of different careers that I can go into.” (A)*
	*“I tried out the program and I love it, and this [STEM] is actually like what I want to do as a career now …It was a good choice.” (A)*
	*“It was like, oh, learn about what the brain is, what brain matter is, what gray matter is, how the brain functions, and the different parts of the brain. It was really cool to learn that because in school you don’t really learn a lot like that, and I guess it changed me because it fuels my interest(s). I love to know more about stuff … it fueled my curiosity and the entire project … it just fueled my interest(s), and I loved it!” (A)*
	*“You don’t really get insight into the medical world other than through YouTube and Google and all that type of stuff. But being able to walk through and talk to actual people who were in medical school. I was able to talk to anesthesiologists and look inside what they were learning and doing …I thought that was pretty cool because like I said it allowed me to gain insight and secure whether I want to do this or not.” (A)*
**Sense of Belonging and Identity**	*“It was really nice to see people that you can look up to that are like you, and you know, you don* t *see those types ofpeople in your field. So, for someone to be there, it kind of gives you hope that I can do this. I can make it; I can be just like them.” (A)*
	*“I feel growing up there weren’t as many like figures we could relate to* … *now you’re able to talk to someone who is able to understand your struggles and [see] how they’ve been able to deal with it and how they are able to… manage their time and like be successful in their fields despite you know, their backgrounds.”(A)*
**Confidence and Self-Efficacy for Science**	*“My teacher, she is the one who gave me the recommendation, and she honestly thinks this program will help me academically with my science because my science grade has been really high since I started.” (A)*
	*“I feel like this [TSAP] changed me and now I have more confidence!” (A)*
	*“She also was always confident, but I remember when she first met the mentees. She went up to them and was like, this program was so great, and I learned all these things and [was] just talking like she was one of the mentors! Just super confident and like she knew everything about the program. It was just awesome to see her growth because I could not have seen her doing that last year. ”(SM about NPM)*
**Creas for Continued Program Improvement**	*“There were definitely times where I didn’t quite involve the peer mentor enough … you have to think about how to balance that time with their roles so they feel like they can be part of the process too.” (SM)*
	*“I also think it would be pretty cool if we could get medical students or people other than our mentors to share their insight into how they came to be.” (A)*
	*“It would be good to talk with other mentors or groups about your research question and some way to get feedback from folks other than their primary mentor or peer mentor just to get them more comfortable talking about their topic in smaller groups before the large group… Maybe we could scaffold that a little more.” (SM)*

Note. A = Ambassador; NPM = Near-Peer Mentor; SM = Senior Mentor.

## ABBREVIATIONS

MCA: Mentoring Competency Assessment
STEM: Science, Technology, Engineering, and Mathematics
STEM-CIS: STEM Career Interest Survey
TSAP: Teen Science Ambassador Program
URM: Underrepresented Minoritized
